# The Value of Body Weight Measurement to Assess Dehydration in Children

**DOI:** 10.1371/journal.pone.0055063

**Published:** 2013-01-29

**Authors:** Isabelle Pruvost, François Dubos, Emmanuel Chazard, Valérie Hue, Alain Duhamel, Alain Martinot

**Affiliations:** 1 Univ Lille Nord de France, UDSL, Lille, France; 2 EA2694, Public Health, Epidemiology and Quality of Care, Lille, France; 3 Paediatric Emergency and Infectious Diseases Unit, CHU Lille, Lille, France; 4 Department of Biostatistics, CHU Lille, Lille, France; The Ohio State Unversity, United States of America

## Abstract

Dehydration secondary to gastroenteritis is one of the most common reasons for office visits and hospital admissions. The indicator most commonly used to estimate dehydration status is acute weight loss. Post-illness weight gain is considered as the gold-standard to determine the true level of dehydration and is widely used to estimate weight loss in research. To determine the value of post-illness weight gain as a gold standard for acute dehydration, we conducted a prospective cohort study in which 293 children, aged 1 month to 2 years, with acute diarrhea were followed for 7 days during a 3-year period. The main outcome measures were an accurate pre-illness weight (if available within 8 days before the diarrhea), post-illness weight, and theoretical weight (predicted from the child’s individual growth chart). Post-illness weight was measured for 231 (79%) and both theoretical and post-illness weights were obtained for 111 (39%). Only 62 (21%) had an accurate pre-illness weight. The correlation between post-illness and theoretical weight was excellent (0.978), but bootstrapped linear regression analysis showed that post-illness weight underestimated theoretical weight by 0.48 kg (95% CI: 0.06–0.79, p<0.02). The mean difference in the fluid deficit calculated was 4.0% of body weight (95% CI: 3.2–4.7, p<0.0001). Theoretical weight overestimated accurate pre-illness weight by 0.21 kg (95% CI: 0.08–0.34, p = 0.002). Post-illness weight underestimated pre-illness weight by 0.19 kg (95% CI: 0.03–0.36, p = 0.02). The prevalence of 5% dehydration according to post-illness weight (21%) was significantly lower than the prevalence estimated by either theoretical weight (60%) or clinical assessment (66%, p<0.0001).These data suggest that post-illness weight is of little value as a gold standard to determine the true level of dehydration. The performance of dehydration signs or scales determined by using post-illness weight as a gold standard has to be reconsidered.

## Introduction

Dehydration secondary to gastroenteritis is one of the most common reasons for office visits and hospital admissions in developed countries [1]–[3]. Among European children <3 years of age, incidence of diarrhea ranges from 0.5 to 1.9 episodes per child per year [4]. The management of acute diarrhea in children is largely based on clinical examination which allows assessment of hydration status. Underestimation of dehydration increases morbidity and mortality, while overestimation can result in inappropriate care and public expenditure. Dehydration is difficult to diagnose clinically [5]. Combinations of examination signs perform markedly better than any individual sign in predicting dehydration [6]. Many scores have been developed to estimate dehydration, but only one, the Clinical Dehydration Scale, has been validated to predict a longer length of Emergency Department (ED) stay and the need for intravenous fluid rehydration [7].

The development and validation of a dehydration scale requires the use of a gold standard. Weight loss is considered as the reference to diagnose dehydration in clinical practice and research [6]. Because the child’s pre-illness weight is rarely known in the acute care setting, post-illness weight gain is widely used to estimate weight loss in research [5]–[13]. A systematic review found that the difference between post-illness (rehydration) weight and acute weight divided by post-illness weight was the best available gold standard to assess the percentage of volume lost [5]. This conclusion, however, was based on only one study, which demonstrated an excellent correlation between pre- and post-illness weight in only 19 children (for 17 of whom pre-illness weight was predicted from growth charts; only 2 had an accurate pre-illness weight) [6]. Other studies that subsequently applied this gold standard have not challenged its correlation with the theoretical weight determined from the growth charts. Moreover, the precise day used as the reference to determine stable post-illness weight was not validated and differed between studies [6]–[10].

The aim of this study was to estimate the value of post-illness weight gain prospectively as a gold standard for acute weight loss in a larger population. Additionally, we sought to study the concordance of the evaluation of 5% dehydration by post-illness weight (gold standard), “theoretical” weight, pre-illness weight, and clinical assessment.

## Methods

### Ethics

The study protocol was approved by the French National Data Protection Authority (Commission Nationale Informatique et Libertés, CNIL) and the French National Institutional Review Board (Comité consultatif sur le traitement de l’information en matière de recherche dans le domaine de la santé, CCTIRS). Parents provided written informed consent before enrolment.

### Study Population

This work was a prospective, observational, cohort study conducted from December 2005 through June 2009 at a tertiary care pediatric ED of a French university hospital with approximately 25,000 annual visits and 4,300 short-stay hospitalizations. This study followed all “STrengthening the Reporting of OBservational studies in Epidemiology” statements [14].

Children aged 1 to 24 months admitted to the ED with a chief complaint of acute diarrhea during the study period, whose parents agreed to study participation and to daily weight surveillance for 7 days following the ED visit, were included. For convenience reasons, recruitment took place during weekday working hours (8 am to 6 pm), to limit the number of investigators. Exclusion criteria were those concerning children with a fluid balance that could be modified by an underlying condition: a chronic disease (cardiac, gastrointestinal, or renal, diabetes mellitus, congenital adrenal hyperplasia, or cystic fibrosis), malnutrition or failure to thrive, and an ileostomy. We also excluded children living more than 30 kilometres away from the hospital, because of our inability to deliver an electronic scale to the home. Children were then treated by the ED staff, regardless of their participation in the study.

### Definitions

Acute diarrhea was defined, ***according to the European Society of Pediatric Gastroenterology and Nutrition guidelines, as illness that started*** <7 days before admission and involved ≥3 soft and/or liquid stools within 24 hours [4]. To be considered accurate, pre-illness weight was defined as a weight of a baby undressed and without diapers, measured within 8 days before the ED visit and before any digestive complaint. No standardization was required for this weight [6].

Theoretical weight was defined as the non-sick baseline weight predicted on the date of the ED visit by extrapolation from the child’s individual growth chart when available (part of French child health passports, which are portable paper records). This extrapolation was considered acceptable when at least three weight points were plotted over time to construct a growth curve and when that curve did not cross percentile lines (to detect any alterations from physiologic growth) [6]. Triage nurses weighed babies, undressed and without diapers, on an electronic scale (SECA, Germany, model 3767021094). This weight was defined as the admission weight. Post-illness weight was defined as the first weight with less than 1% of differences between two consecutive daily weight measurements, after diarrhea and vomiting had disappeared**.** Dehydration was defined as a fluid deficit of 5% or greater. It was calculated as (post-illness weight – admission weight)/(post-illness weight) x 100 for the “gold standard” method, or as (theoretical weight – admission weight)/(theoretical weight) x 100 or (pre-illness weight – admission weight)/(pre-illness weight) x 100. The presence of moderate or severe dehydration was also estimated by the physician, based on clinical assessment of the different signs of dehydration.

At the time of discharge from the ED, all included patients, whether admitted to the hospital or discharged home, were enrolled for a follow-up visit. Naked body weight was measured by parents on same regularly calibrated scale as at admission, and children were weighed daily at home in the morning on a similarly calibrated electronic scale, before the first meal, for seven days in a row. These weights were recorded daily on a questionnaire with the last day of diarrhea or vomiting.

### Data Analysis

Data were entered into Epi-Data 2.1b software with check-in controls (Epidata Association, Odense, Denmark). Results were expressed as means, medians, and either standard deviations (SD) or interquartile ranges according to their distribution for continuous variables, and frequencies and percentages for categorical variables. The 95% confidence intervals (CIs) were calculated. The relationship between theoretical and post-illness weights was assessed by the Pearson correlation coefficient. A linear regression was performed to explain theoretical weight by post-illness weight. The CIs for the intercept and the regression coefficient were determined with the bootstrap method [15]. This method provides robust estimates for CIs without making any assumptions about the distribution of the characteristics. As a high correlation does not automatically imply that there is a good agreement between two methods, the agreement between these two weights was evaluated by Bland-Altman plots [16].

The correlation between the accurate pre-illness weight and each of the theoretical and the post-illness weights were investigated with a linear regression analysis and the intraclass correlation coefficient. These different weights were compared with a paired student’s t-test and a linear regression analysis. All statistical analyses were performed with SAS software (SAS Institute, Cary, NC). A p value less than 0.05 was considered statistically significant.

## Results

### Patient Characteristics

A total of 293 children (median age: 12 months, mean: 11.5±6.2 SD, 57% boys) were included in the study for follow-up. Of those, 14% (n  = 40) were admitted to the hospital, 30% (n  = 88) stayed for a few hours in the observation unit for intravenous rehydration and 56% (n  = 165) were directly discharged home from the ED. The median duration of diarrhea before admission was 3 days (IQR: 1–4), and 91% (n  = 266) of the patients had vomiting. The mean weight at admission was 9.0 kg ±2.1 SD, 61% (n  = 179) of the patients had a central temperature >38°C at home and 27% (n  = 79) had no previous medical visit for this episode of diarrhea before admission.

**Figure 1:**
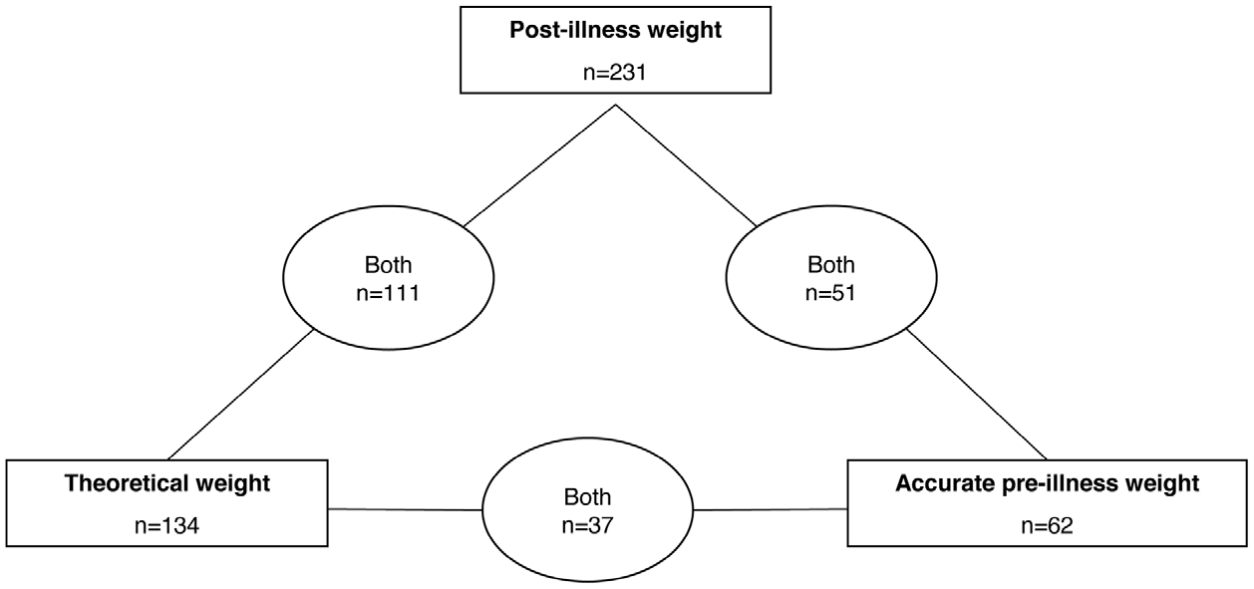
Distribution of children with acute diarrhea by available methods of measure of the weight (n = 293). **Pre-illness weight**: measured within 8 days before the ED visit. **Theoretical weight**: predicted from the child’s individual growth chart. **Post-illness weight**: the first weight when two consecutive daily weight measurements differed by <1%, after diarrhea and vomiting had disappeared.

Figure 1 describes the different subgroups based on available weight measurements. Of the 293 patients, 21% (n  = 62) had been weighed within 8 days before the ED visit, before any digestive complaint. Theoretical weight could be measured for only 46% (n  = 134) of the patients, because 41% (n  = 119) of patients had been brought to the ED without child health passports, and 14% (n  = 40) did not have valid growth charts to determine the theoretical weight as defined in the methods. Post-illness weight was measured for 79% (n  = 231) of patients; 20% (n  = 58) were excluded from the analysis, either because they did not return the weight questionnaire or were not weighed for 7 consecutive days, and 1% (n  = 4) because they did not reach a stable weight within 8 days of ED visit.

### Value of the Post-illness Weight Gain as a Gold Standard for Estimating Acute Weight Loss and Identifying Dehydration

**Figure 2:**
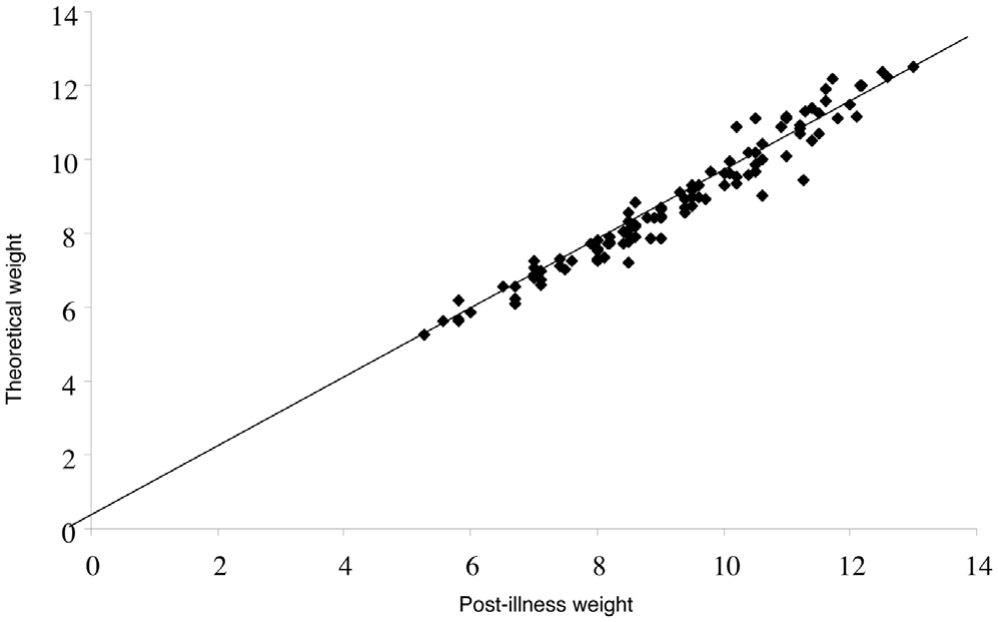
Correlation between theoreti cal and post-illness weight, assessed by Pearson Correlation (n  = 111). Postillness weight  = 0.978 x Theoretical weight (p<10^−6^).

**Figure 3:**
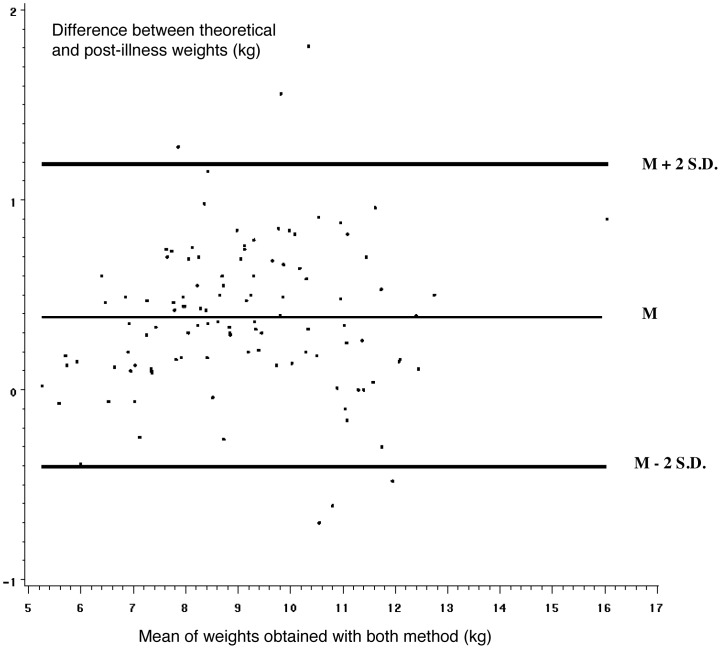
Agreement between theoretical and post-illness weight, analysed with a Bland-Altman plot, n = 111. M: mean, SD: Standard Deviation. Mean difference, which is the best guess as to the “correct” result. 95% limits of agreement. When the agreement between two measures is high, the mean difference between these two measures is close to 0. The 95% limits of agreement permit visual judgement of how well two methods of measurement agree. The smaller the range between these two limits the better the agreement is.

Both post-illness and theoretical weights were obtained for 111 children (Figure 1). The mean age of this subgroup was 11.7 months ±5.9 SD, (median = 13.0). The correlation between post-illness and theoretical weight was excellent, with a Pearson correlation coefficient of 0.978 (Figure 2). The statistical assessment of concordance between these two weights measured by the intraclass correlation coefficient was also excellent (0.963). A linear regression analysis, to explain the theoretical weight by the post-illness weight was performed. With this method, the regression coefficient determined with the bootstrap method was 0.99 (95% CI: 0.95–1.04) and post-illness weight underestimated theoretical weight by 0.48 kg (95% CI: 0.06–0.79, p<0.02). Figure 3 shows the agreement between these two weights analyzed with a Bland-Altman plot that showed a pretty scattered distribution. Mean theoretical weight was 9.26 kg ±1.91 SD, while the mean post-illness weight was 8.88 kg ±1.87 SD. The mean difference between these two weights was 0.38 kg (95% CI: 0.30–0.45, p<0.0001). The mean difference between the fluid deficit calculated from the theoretical weight and that based on post-illness weight was 4.0% of body weight (95% CI: 3.2–4.7%, p<0.0001).

The prevalence of 5% dehydration determined by post-illness weight was 21% versus 60% by theoretical weight (p<0.001). The prevalence of moderate or severe dehydration by clinical assessment was 66%, also significantly different from the prevalence determined by post-illness weight (p<0.001).

### Value of the Accurate Pre-illness Weight

Both post-illness and accurate pre-illness weights were obtained for 51 children (17%) (Figure 1) who did not differ significantly from others in terms of age (mean: 11.9±6.1 SD vs. 13.4±6.3 SD; p  = 0.1), post-illness weight (mean: 8.9±1.9 SD vs. 9.1±2.0 SD; p  = 0.4), and theoretical weight (mean: 9.0±1.9 SD vs. 9.7±2.1 SD; p  = 0.1). There was an excellent correlation between post-illness and pre-illness weight, with a Pearson correlation coefficient of 0.979 (p<0.0001). Post-illness weight underestimated pre-illness weight by 0.19 kg (95% CI: 0.03–0.36, p = 0.02).

Both accurate and theoretical pre-illness weights were obtained for only 37 children (13%) (Figure 1). These children did not differ significantly from others in terms of age (mean: 12.0±6.2 SD vs. 13.3±5.8 SD; p  = 0.2), post-illness weight (mean: 8.8±1.9 SD vs. 9.3±2.2 SD; p  = 0.2), and theoretical weight (mean: 9.1±1.9 SD vs. 9.5±2.2 SD; p  = 0.3). There was an excellent correlation between theoretical and accurate pre-illness weight, with a Pearson correlation coefficient of 0.985. Theoretical weight overestimated accurate pre-illness weight by 0.21 kg (95% CI: 0.08–0.34, p = 0.002).

## Discussion

In our study, post-illness weight gain was not a gold standard for estimating acute weight loss and identifying dehydration in children with acute gastroenteritis. The mean absolute difference between fluid deficit calculated from the theoretical weight and that calculated from the post-illness weight was 4% of body weight (95% CI: 3.2–4.7, p<0.0001). As shown by the Bland-Altman plot, the agreement between these two weights was poor and post-illness weight underestimated dehydration. The prevalence of 5% dehydration according to post-illness weight was significantly lower than the prevalence estimated by either theoretical weight or by the physician’s clinical assessment.

Gorelick et al. showed an excellent correlation between weight measured after treatment for children with acute gastroenteritis and their pre-illness “well” weight [6]. There was however a small positive intercept of the regression line, which suggests a slight bias towards an underestimation of pre-illness weight by post-illness weight, but without either a statistically significant or a clinically meaningful difference in dehydration between the two methods of measurement (0.67%). The differences between our results and those obtained using this so-called gold standard may stem from the methods used to determine final weight and from the very small number of patients to validate this standard (n = 19) [6]. Our study, which included more than five times more patients (n = 111) and applied the bootstrap method to obtain robust estimations for the confidence intervals, did not confirm that the post-illness weight is an appropriate gold standard for evaluating 5% dehydration in children with acute gastroenteritis. Mackenzie et al. [10] also found that post-illness weight overestimated dehydration by a mean of 3.2%, perhaps because objective clinical signs of dehydration tend to appear when fluid deficit is less than 5%. Underestimation of dehydration by post-illness weight might be due in part to weight loss from catabolism and nutritional loss, not yet offset at the end of diarrhea.

Our method of determining post-illness weight (two consecutive daily weight measurements that differed by <1%, after diarrhea and vomiting had disappeared) resulted in a smaller margin of error than in other studies, where post-treatment weights were considered stable if they agreed to within ±2% at two consecutive but not daily measures [6], [11], [13]. The precise day used as reference to determine the stable rehydrated weight has not yet been validated and differs between studies [6]–[10]. Indeed Mackenzie et al. defined post-illness weight as the naked weight at 24 or 48 hours after admission, with no clinical signs of dehydration and biochemical results back to normal [10]. Others weighed patients at follow up visits every 48–72 hours until a “stable weight” was reached. Patients not fully rehydrated within 2 weeks of the ED visit were excluded from the research [13]. Most studies have also used additional assessments to reinforce their choice of a true post-illness weight [5]. For example, Teach et al. [17] used the weight at normalization of examination findings with low urine specific gravity. Incorporating other assessments not based on weight into the gold standard could theoretically bias the results. Steiner et al. showed that varying the date of the final rehydration weight could introduce distortions [5]. For example, if it is obtained too early, children may still be dehydrated or may be overhydrated because of aggressive intravenous fluid administration.

Our study has limitations. Because significant dehydration is uncommon among children with gastroenteritis in industrialized countries, the number of children with dehydration in this study was relatively small and thus led to less precise estimates. However, the use of bootstrap methods limited imprecision. Another limitation was that we did not provide any standardization for “pre-illness weight” because it was measured before inclusion. Weights during well child care are sometimes not naked weight, which could overestimate pre-illness weight. The determination of acute weight loss by the theoretical weight, extrapolated from growth charts, most probably leads to bias, as children’s growth is irregular. Overestimation of dehydration by theoretical weight might also be partially due to the relative imprecision of growth charts. Furthermore, theoretical weight could only be measured for 49% of the patients in our study. Finally, the use of body weight to determine the percentage of dehydration might also be criticized: 70% of the body is made of water, and acute weight loss is mainly due to acute water loss. But the same weight loss could over- or underestimates water loss because of variations in the digestive water retention or because the child was weighed before or after passing stool, vomiting, or just after feeding. All of these could produce a difference of up to 100 or 200 g, which represents roughly up to 3% of a 6-kg child.

The current study’s findings suggest that post-illness weight gain is not a gold standard in research for estimating acute weight loss and identifying dehydration in children aged 1 to 24 months with acute gastroenteritis. Post-illness weight depends on rehydration and also on nutritional status with large individual variations between these two components. Thus this figure does not permit to estimate fluid loss based on post-illness weight. The results of many previous studies that provided diagnostic value of dehydration signs or scales based on this so-called “post-illness weight” gold standard have to be reconsidered. Future studies should focus on evaluating only children with accurate recent pre-illness weight for research purpose, although its availability (21% of our patients weighed in the 8 days before the ED visit and before any digestive disturbances) and reliability limit its use in current practice.

## References

[1] Parashar UD, Hummelman EG, Bresee JS, Miller MA, Glass RI (2003) Global illness and deaths caused by rotavirus disease in children. Emerg Infect Dis 9: 565–72. 12737740 10.3201/eid0905.020562 PMC2972763

[2] Soriano-Gabarro M, Mrukowicz J, Vesikari T, Verstraeten T (2006) Burden of rotavirus disease in European Union Countries. Pediatr Infect Dis J 25: S7–S11. 16397431 10.1097/01.inf.0000197622.98559.01

[3] Glass RI, Lew JF, Gangarosa RE, LeBaron CW, Ho MS (1991) Estimates of morbidity and mortality rates for diarrheal diseases in American children. J Pediatr 118: S27–33. 2007954 10.1016/s0022-3476(05)81422-2

[4] Guarino A, Albano F, Ashkenazi S, Gendrel D, Hoekstra JH, et al (2008) European Society for Paediatric Gastroenterology, Hepatology, and Nutrition/European Society for Paediatric Infectious Diseases evidence-based guidelines for the management of acute gastroenteritis in children in Europe. J Pediatr Gastroenterol Nutr 46: S81–122. 18460974 10.1097/MPG.0b013e31816f7b16

[5] Steiner MJ, De Walt DA, Byerley JS (2004) Is this child dehydrated? JAMA 291: 2746–54. 15187057 10.1001/jama.291.22.2746

[6] Gorelick MH, Shaw KN, Murphy KO (1997) Validity and reliability of clinical signs in the diagnosis of dehydration in children. Pediatrics 99: 99–106. 10.1542/peds.99.5.e6 9113963

[7] Friedman JN, Goldman RD, Srivastava R, Parkin PC (2004) Development of a clinical dehydration scale for use in children between 1 and 36 months of age. J Pediatr 145: 201–7. 15289767 10.1016/j.jpeds.2004.05.035

[8] Vega RM, Avner JR (1997) A prospective study of the usefulness of clinical and laboratory parameters for predicting percentage of dehydration in children. Pediatr Emerg Care 13: 179–82. 9220501 10.1097/00006565-199706000-00001

[9] Duggan C, Refat M, Hashem M, Wolff M, Fayad I, et al (1996) How valid are clinical signs of dehydration in infants? J Pediatr Gastroenterol Nutr 22: 56–61. 8788288 10.1097/00005176-199601000-00009

[10] Mackenzie A, Barnes G, Shann F (1989) Clinical signs of dehydration in children. Lancet 2: 605–7. 2570294 10.1016/s0140-6736(89)90723-x

[11] Saavedra JM, Harris GD, Li S, Finberg L (1991) Capillary refilling (skin turgor) in the assessment of dehydration. Am J Dis Child 145: 296–8. 2003478 10.1001/archpedi.1991.02160030064022

[12] Yilmaz K, Karaböcüoglu M, Citak A, Uzel N (2002) Evaluation of laboratory tests in dehydrated children with acute gastroenteritis. J Paediatr Child Health 38: 226–8. 12047687 10.1046/j.1440-1754.2002.00792.x

[13] Porter SC, Fleisher GR, Kohane IS, Mandl KD (2003) The value of parental report for diagnosis and management of dehydration in the emergency department. Ann Emerg Med 41: 196–205. 12548269 10.1067/mem.2003.5

[14] von Elm E, Altman DG, Egger M, Pocock SJ, Gøtzsche PC, Vandenbroucke JP, et al (2007) The Strengthening the Reporting of Observational Studies in Epidemiology (STROBE) statement: guidelines for reporting observational studies. PLoS Med 4: e296. 17941714 10.1371/journal.pmed.0040296 PMC2020495

[15] Carpenter J, Bithell J (2000) Bootstrap confidence intervals: when, which, what? A practical guide for medical statisticians. Stat Med 19: 1141–64. 10797513 10.1002/(sici)1097-0258(20000515)19:9<1141::aid-sim479>3.0.co;2-f

[16] Bland JM, Altman DG (1986) Statistical methods for assessing agreement between two methods of clinical measurement. Lancet 1: 307–10. 2868172

[17] Teach SJ, Yates EW, Feld LG (1997) Laboratory predictors of fluid deficit in acutely dehydrated children. Clin Pediatr (Phila) 36: 395–400. 9241476 10.1177/000992289703600703

